# Association of Glucose-6-Phosphate Dehydrogenase Deficiency With Outcomes in US Veterans With COVID-19

**DOI:** 10.1001/jamanetworkopen.2023.5626

**Published:** 2023-03-29

**Authors:** Sarah H. Elsea, Javad Razjouyan, Kyung Min Lee, Julie A. Lynch, Sharyl Martini, Lavannya M. Pandit

**Affiliations:** 1 Center for Translational Research on Inflammatory Diseases (CTRID), Michael E. DeBakey Veterans Affairs Medical Center, Houston, Texas; 2 Department of Molecular and Human Genetics, Baylor College of Medicine, Houston, Texas; 3 Big Data Scientist Training Enhancement Program (BD-STEP), VA Office of Research and Development, Salt Lake City, Utah; 4 VA Informatics and Computing Infrastructure, VA Salt Lake City Health Care System, Salt Lake City, Utah; 5 Department of Internal Medicine, School of Medicine, University of Utah, Salt Lake City; 6 National Neurology Program, Specialty Care Program Office, Veterans Health Administration, Washington, DC; 7 Department of Neurology, Baylor College of Medicine, Houston, Texas; 8 Sleep Section, Department of Medicine-Pulmonary Critical Care, Baylor College of Medicine, Houston, Texas

## Abstract

**Importance:**

The underlying biological risk factors for severe outcome due to SAR-CoV-2 infection are not well defined.

**Objective:**

To determine the association between glucose-6-phosphate dehydrogenase (G6PD) deficiency and severity of COVID-19.

**Design, Setting, and Participants:**

This retrospective cohort study included analysis of 24 700 veterans with G6PD enzyme testing prior to January 1, 2020, obtained through the US Veterans Health Administration national databases. These veterans were cross-referenced with the Veterans Administration COVID-19 Shared Data Resource for SARS-CoV-2 testing from February 15, 2020, to January 1, 2021. The final study population consisted of 4811 veterans who tested positive for SARS-CoV-2. Statistical analysis was performed from June to December 2021.

**Exposures:**

G6PD deficiency.

**Main Outcomes and Measures:**

COVID-19 severe illness, as defined by the Centers for Disease Control and Prevention: hospitalization, need for mechanical ventilation and/or intensive care unit admission, or in-hospital mortality after a positive SARS-CoV-2 test.

**Results:**

Among 4811 veterans in the Veterans Health Administration who had historical G6PD enzyme activity test results and SARS-CoV-2 positivity included in this study, 3868 (80.4%) were male, 1553 (32.3%) were Black, and 1855 (39%) were White; 1228 (25.5%) were 65 years or older and 3583 (74.5%) were younger than 65 years. There were no significant differences in age, body mass index, or Charlson Comorbidity Index were present between the veterans with G6PD deficiency and without G6PD deficiency. Among these veterans with SARS-CoV-2 infection, G6PD deficiency was more prevalent in Black male veterans (309 of 454 [68.1%]) compared with other racial and ethnic groups. Black male veterans less than 65 years of age with G6PD deficiency had approximately 1.5-fold increased likelihood of developing severe outcomes from SARS-CoV-2 infection compared with Black male veterans without G6PD deficiency (OR, 1.47; 95% CI, 1.03-2.09). In the small subset of White male veterans with G6PD deficiency, we observed an approximately 3.6-fold increased likelihood of developing severe outcomes from SARS-CoV-2 infection compared with White male veterans aged 65 years or older without G6PD deficiency (OR, 3.58; 95% CI, 1.64-7.80). This difference between veterans with and without G6PD deficiency was not observed in younger White male veterans or older Black male veterans, nor in smaller subsets of other male veterans or in female veterans of any age.

**Conclusions and Relevance:**

In this cohort study of COVID-19–positive veterans, Black male veterans less than 65 years of age and White male veterans 65 years of age or older with G6PD deficiency had an increased likelihood of developing severe COVID-19 compared with veterans without G6PD deficiency. These data indicate a need to consider the potential for G6PD deficiency prior to treatment of patients with SARS-CoV-2 infection as part of clinical strategies to mitigate severe outcomes.

## Key Points

**Question:**

Is G6PD deficiency, the most common enzyme deficiency in the world, associated with COVID-19 severity in US veterans?

**Findings:**

In this cohort study of 24 700 veterans, G6PD deficiency was present in 9.4% of veterans with SARS-CoV-2 infection and was associated with a 1.5-fold increased likelihood of severe outcomes in male veterans less than 65 years of age who self-identified as Black, and a 3.6-fold greater likelihood of severe outcomes in male veterans 65 years of age and older who self-identified as White.

**Meaning:**

These results suggest that G6PD deficiency was more prevalent in minoritized racial and ethnic communities and associated with increased likelihood for severe outcomes due to SARS-CoV-2 infection, supporting the need for additional investigations to identify individuals at greatest risk and to define the best approaches for therapeutic intervention.

## Teaser

This cohort study examines whether there is an association between glucose-6-phosphate dehydrogenase (G6PD) deficiency and severity of COVID-19 outcomes among US veterans.

## Introduction

SARS-CoV-2 has devastated the global community with almost 6 million deaths and expected ongoing deaths with the rise of new variants.^1,2^ One challenging aspect of treating COVID-19 is understanding why certain infected individuals experience severe, life-threatening complications, while others remain minimally symptomatic. Severity risks associated with SARS-CoV-2 infection are purported to be related to variable factors including patient ancestry, socioeconomic status, health care utilization, and comorbid conditions.^3,4,5^ Although socioeconomic status and social determinants of health have been discussed as possible causes for the higher burden of increased morbidity and mortality in minoritized racial and ethnic communities in the US, the contribution of biology toward disease severity has been minimally explored.^6^ As a result, despite almost 3 years of observational data, the biologic bases of susceptibility to SARS-CoV-2 infection and risks of severe outcomes after infection are largely unknown.

Viral infection triggers massive reactive oxidative species production and oxidative damage. Glutathione (GSH) is essential and protects the body from the harmful effects of oxidative damage from excess reactive oxygen radicals.^5^ Glucose-6-phosphate dehydrogenase (G6PD) is necessary to prevent the exhaustion and depletion of cellular GSH. G6PD deficiency is a genetic metabolic abnormality and is the most common enzyme deficiency, affecting more than 400 million people worldwide.^7^ Although this X-linked condition is more commonly described in men, hemizygous male individuals and heterozygous female individuals may be affected. In the US, G6PD deficiency has an estimated prevalence of 10% to 14% among Black men.^8,9^ Acquired deficiency of G6PD is associated with obesity and diabetes, as G6PD enzyme activity is moderated by hyperglycemia, and epidemiological evidence suggests that patients with G6PD deficiency have a higher risk of developing diabetes.^5,10,11^ A recent study highlighted associations with G6PD deficiency and cardiovascular risk, including hypertension and cardiomyopathy.^12^ Although the majority of individuals with G6PD deficiency are asymptomatic, a trigger (food, medication, or infection) may lead to hemolytic anemia, hemoglobinuria, and hematuria. Individuals with inherited or acquired G6PD deficiency are vulnerable to oxidative stress and heightened susceptibility to microbial infection.^13^ Recent publications have outlined several pieces of evidence suggesting that G6PD deficiency may increase susceptibility to, and severity of, COVID-19.^5,14,15,16,17,18,19^ Further supporting this theory are ex vivo studies in G6PD-deficient cells showing increased susceptibility to infection and cell death by human coronavirus HCoV-229E infection and reduced NF-κB activation in coronavirus-infected G6PD-deficient cells.^20,21^ Additionally, multiple studies demonstrate that diabetes is independently associated with COVID-19 severity and increased mortality.^22,23,24^ The compounded effects of diabetes, hyperglycemia, and acquired or inherited G6PD deficiency may increase the susceptibility of patients with G6PD deficiency to worse outcomes from COVID-19.

G6PD status is already known for all active military members and many veterans since the US Department of Defense (DOD) has mandated all US Army personnel undergo testing for G6PD deficiency at the time of entry into service.^25^ The prevalence of G6PD deficiency within the Armed Forces among non-Hispanic Black male individuals and female individuals is 15.9% compared with 2.2% overall.^25^

This study examined whether the presence of G6PD deficiency in veterans diagnosed with SARS-CoV-2 infection was associated with increased odds of developing severe COVID-19 compared with veterans without G6PD deficiency. Our primary objectives were to report the prevalence of G6PD deficiency among veterans with COVID-19 and to measure the severity of illness (mortality, hospitalization, need for ventilator support, and intensive care unit admission) among those veterans with COVID-19. The purpose of this analysis was to determine if an association exists between the development of severe COVID-19 and G6PD deficiency in US veterans. We also examined whether an association between G6PD deficiency and COVID-19 severity differed by age, sex, and race with consideration of common comorbidities.

## Methods

This cohort study was approved by the Baylor College of Medicine institutional review board and Department of Veterans Affairs with a local waiver of consent. Local waiver of consent was granted given the large number of veterans in the database and the preference to keep the individual veteran data anonymous for patient privacy protection. This study followed the Strengthening the Reporting of Observational Studies in Epidemiology (STROBE) reporting guideline.

### Study Design, Setting, and Participants

**Figure 1.**
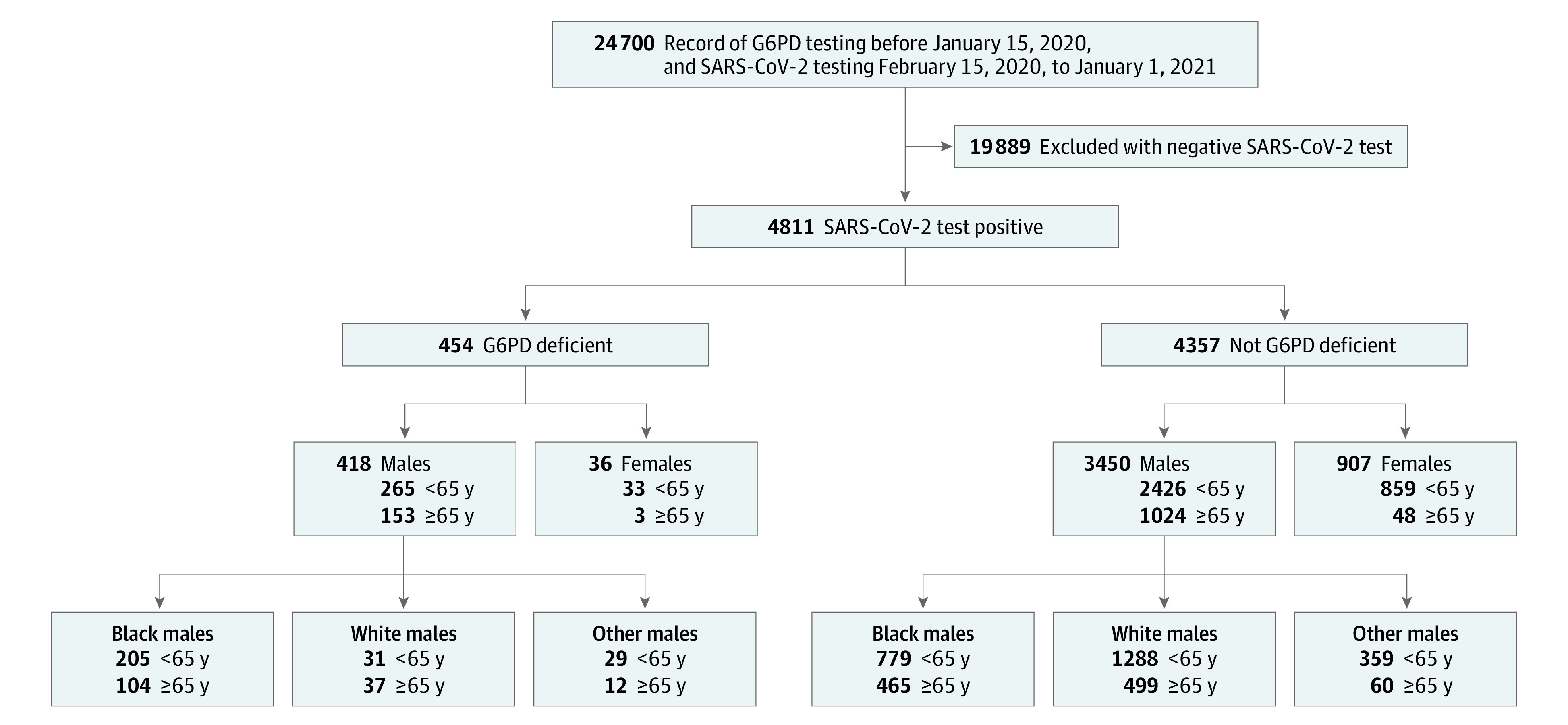
Study Population of SARS-CoV-2–Positive US Veterans With or Without G6PD Deficiency US veterans with both a prior medical record for G6PD deficiency testing and SARS-CoV-2 testing between February 15, 2020, to January 1, 2021, are shown (n = 24 700). The SARS-CoV-2–positive subgroup (n = 4811) was stratified by sex (male, female) and self-identified race (Black, White, other [self-identified as neither Black nor White or self-identified as Asian, Pacific Islander, or American Indian or Alaska Native]) for further study assessment.

Data supporting this retrospective cohort study were accessed through the national electronic health record (EHR) of veterans enrolled in the Veterans Health Administration (VHA) as active health care users in the 2 years before the study period and defined as those who had received any primary care encounter within VHA facilities including outpatient mental health, screening health, pharmacy, radiology, or laboratory services. Veterans in the VHA who had historical G6PD enzyme activity test results, as standard protocol per the DOD, were further cross-referenced for positive SARS-CoV-2 testing through the VA COVID-19 Shared Data Resource^26^ (CSDR) (n = 24 700) (Figure 1). The CSDR includes demographic and clinical information related to COVID-19 for all patients tested for SARS-CoV-2 within VHA or whose positive test result outside VHA was recorded in VHA clinical notes.^14,27^ This study analyzed EHR data for veterans in the VHA with historical G6PD test results who had a positive molecular PCR-SARS-CoV-2 test or historical positive test in VHA clinical notes from February 15, 2020, to January 1, 2021 (n = 4811) (Figure 1). Because the earliest SARS-CoV-2 testing date reported in the CSDR was February 16, 2020, we considered any veteran alive as of February 15, 2020, as eligible for SARS-CoV-2 testing. Although EHR limitations prevent the authors from ascertaining reasons for SARS-CoV-2 testing, which could lend potential biases, the veterans under study who received testing were actively receiving care within the VHA and had equal access to care and testing at all VHA facilities. All data included in this study were generated prior to the widespread availability of SARS-CoV-2 screening of asymptomatic individuals, at-home testing, or vaccination to the veteran population.

In this study, we used self-identified sex (male or female) and self-identified race (White, Black, or other [self-identified as Asian, Pacific Islander, American Indian or Alaska Native]) to stratify all included veterans within the VHA. Due to the small number within each of the race categories that were not Black nor White, they were considered as a single category because no clear conclusions could be made of any individual race in these groups. Any missing values were assigned as unknown, with exceptions being age (data excluded) and body mass index (BMI [calculated as weight in kilograms divided by height in meters squared]) for which a median BMI was assigned. No sensitivity analysis was performed.

### Variable Exposure and Outcomes

G6PD deficiency as the primary variable was determined by quantitative enzyme activity testing, as performed and reported at various VHA US laboratories any time prior to January 1, 2020, with results obtained from the VHA Corporate Data Warehouse, a data repository of national EHR data of all individuals who received care in the VHA. Any veteran who ever had a value falling below the testing laboratory’s reference range was classified as G6PD-deficient. Although the authors acknowledge the challenge of confirming G6PD enzyme test results within the study population, this clinical test was performed at Clinical Laboratory Improvement Amendments–accredited laboratories and rigorously applied throughout the veteran population through protocols established through the DOD.^18^ The primary outcome measure was COVID-19 severe illness, defined as any of the following clinical scenarios occurring after a positive SARS-CoV-2 test: hospitalization, need for mechanical ventilation, intensive care unit admission or transfer, or in-hospital mortality.^28^ For odds ratios (ORs) and all combined analyses with regards to the primary outcome measure of severity of illness, individuals were only counted once toward calculation of the composite score.

### Covariates

To address potential contributions of modifiers and confounders within the study population, we obtained participants’ demographics and comorbidities from the CSDR. Based upon the Centers for Disease Control and Prevention’s published comorbid medical conditions that confer increased severity of illness from COVID-19 (accessed April 15, 2021),^28,29,30^ the following medical conditions as modifiers were extracted and curated from VHA EHR: age (dichotomized into <65 years and ≥65 years), BMI (dichotomized into <30 and ≥30), Charlson Comorbidity Index (dichotomized into <2 and ≥2),^31,32^ self-identified race and sex, and medical history for the presence of the following: diabetes, chronic kidney disease (CKD), coronary atherosclerosis and other heart disease (CAHD), cardiomyopathy, congestive heart failure (CHF), cardiovascular disease including hypertension (CVD), cancer, chronic obstructive pulmonary disease (COPD), human immunodeficiency virus (HIV), chronic liver disease (CLD), cirrhosis, and alcohol dependency, as indicated by *International Statistical Classification of Diseases and Related Health Problems, Tenth Revision (ICD-10)* codes.^29^

### Statistical Analysis

**Figure 2.**
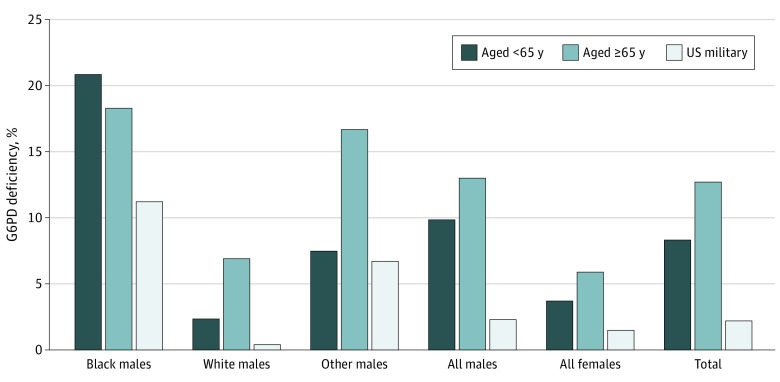
Prevalence of G6PD Deficiency in US Veteran Population That Tested Positive for SARS-CoV-2 Infection Of the total male veterans (n = 3868) in this SARS-CoV-2–positive cohort (n = 4811), 10.8% (n = 418) had G6PD deficiency with the following distribution across racial ancestries: Black male veterans: 19.9% (309 of 1553), White male veterans: 3.7% (68 of 1855), and other male veterans (self-identified as neither Black nor White or self-identified as Asian, Pacific Islander, or American Indian or Alaska Native): 8.9% (41 of 460). Of the total female veterans of all racial ancestries (n = 907) in this SARS-CoV-2 positive cohort, 3.8% (36) had G6PD deficiency. These data show that the prevalence of G6PD deficiency in veterans positive for SARS-CoV-2 was higher than expected in the military population. All SARS-CoV-2 testing performed between February 15, 2020, to January 1, 2021. The percentages of US veterans with G6PD deficiency are presented as reported by the US Department of Defense.^18^

*G6PD* is located on the X chromosome. As an X-linked condition, G6PD deficiency is more prevalent in men, with variable expression in heterozygous female individuals. The prevalence of G6PD deficiency differs significantly among ancestral groups.^8^ Few female veterans and few men of other high-risk ancestries were present in our cohort (Figure 1 and Figure 2; eTable 1 in Supplement 1). For these reasons, we stratified by racial ancestry and sex to define 4 groups: (1) male-Black, (2) male-White, (3) male-other, and (4) female-all. As 80% of COVID-19-associated deaths were among adults aged at least 65 years, we further stratified by age.^28^ We performed descriptive and multivariate analyses to compare baseline characteristics across the 4 sex and racial groups. SAS 9.2 (SAS Institute) and MATLAB R2017b (MathWorks) were used for data preparation and statistical analyses from June to December 2021. Two-tailed hypothesis testing was performed using the significance level of 5%. To test the association between G6PD deficiency and COVID-19 severity, we applied logistic regression models, comparing the frequency of adverse clinical outcomes between veterans with and without G6PD deficiency, adjusting for specific covariates and comorbid conditions previously determined to modify COVID-19 clinical severity, specifically age, diabetes, CKD, and BMI.^33,34^

## Results

There were 24 700 veterans in the VHA who had historical G6PD enzyme activity test results. Among the 4811 veterans who tested positive for SARS-CoV-2 infection included in this study, 3868 (80.4%) were male, 1553 (32.3%) were Black, and 1855 (39%) were White; 1228 (25.5%) were 65 years or older and 3583 (74.5%) were younger than 65 years.

### Comorbidities in Veterans with G6PD Deficiency and SARS-CoV-2 Infection 

**Figure 3.**
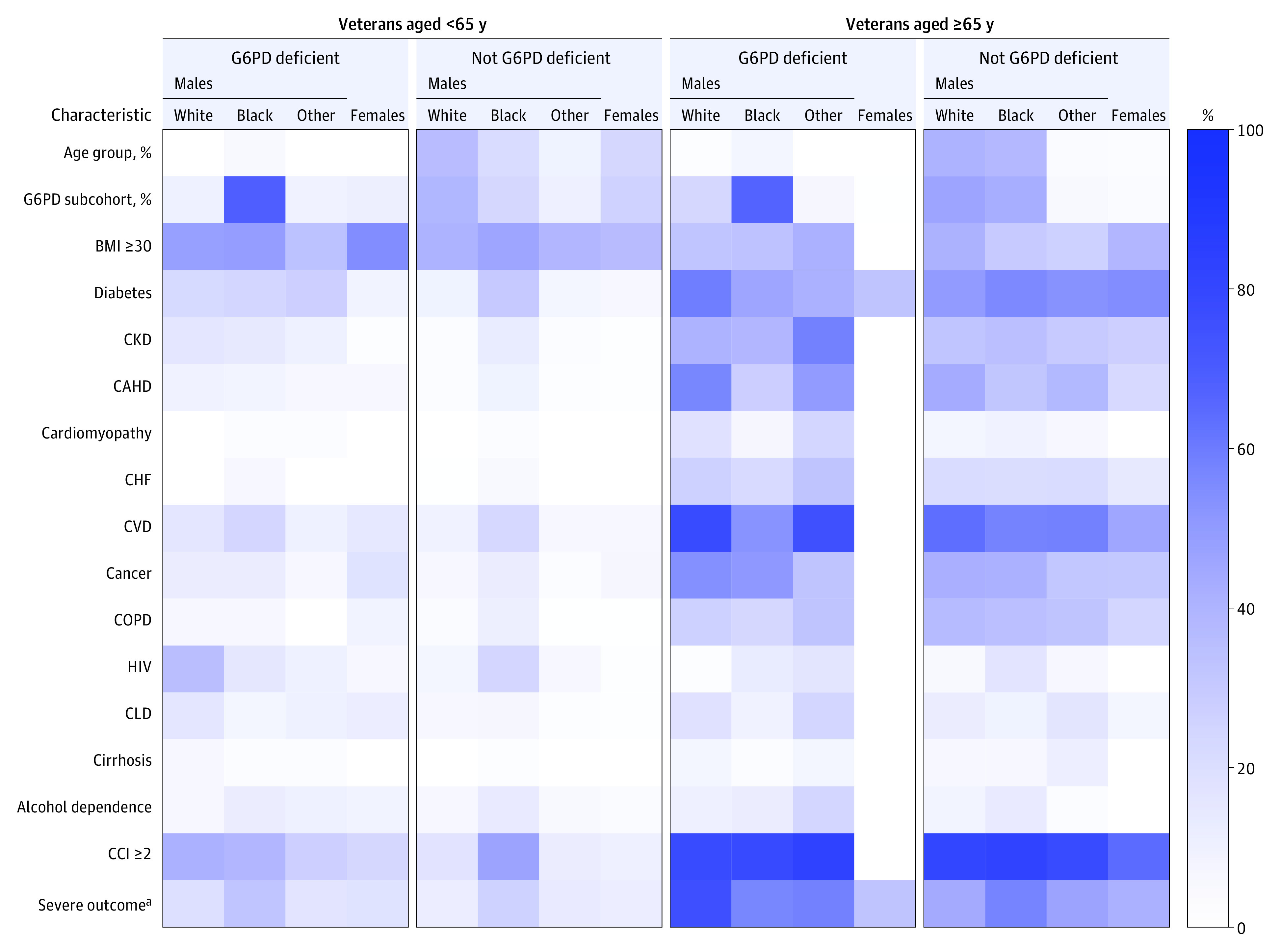
Clinical Characteristics of US Veterans Testing Positive for SARS-CoV-2 With or Without G6PD Deficiency Heatmap illustrates the prevalence of comorbidities commonly associated with risk for severe outcomes due to SARS-CoV-2 infection (n = 4811). Veterans were grouped by age (<65 y and ≥65 y) and self-reported sex and race. Percentages for each comorbidity indicated are represented by the heatmap scale shown on the right. BMI indicates body mass index (calculated as weight in kilograms divided by height in meters squared); CAHD, coronary atherosclerosis and other heart disease; CCI, Charlson Comorbidity Index; CHF, congestive heart failure; CKD, chronic kidney disease; CLD, chronic liver disease; COPD, chronic obstructive pulmonary disease; CVD, cardiovascular disease including hypertension; HIV, human immunodeficiency virus; Other, male veterans with other race (did not self-identify as White or Black, or self-identified as Asian, Pacific Islander, or American Indian or Alaska Native). ^a^Severe outcomes include (1) in-hospital mortality, (2) hospitalization, (3) intensive care unit admission, or (4) mechanical ventilation. All data were extracted as indicated by *International Statistical Classification of Diseases and Related Health Problems, Tenth Revision (ICD-10)* codes.^28^ See eTable 1 in Supplement 1 for detailed information and raw data and eFigure 1 in Supplement 1 for representation of total cohort data.

A total of 4811 veterans who tested positive for SARS-CoV-2 during the study period were subcategorized by G6PD status, sex, race, and age (Figure 1; eTable 1 in Supplement 1). There were no significant differences in age, BMI, or Charlson Comorbidity Index among the G6PD-deficient and non-G6PD-deficient SARS-CoV-2–positive veterans. (Figure 3) (eTable 1 and eFigure 1 in Supplement 1). Male veterans composed the majority of the veterans who were SARS-CoV-2 positive across the entire cohort (80.4% male [3868 of 4811]), with G6PD deficiency documented in 10.8% (418 of 3868). Although the comorbidities present in Black male veterans with or without G6PD deficiency did not differ, several comorbidities were more frequently observed in White male veterans with G6PD deficiency vs the non–G6PD deficiency group, including diabetes, CKD, CAHD, CVD, HIV, CLD, and cirrhosis in White men less than 65 years of age and cardiomyopathy in White men who were at least 65 years of age (Figure 3; eTable 1 in Supplement 1). While some of these associations, including diabetes and hypertension, have been previously reported,^10,35,36,37,38^ the underlying etiology and possible association with G6PD deficiency with each of these clinical findings requires further investigation.

Given that G6PD deficiency is X-linked, only 3.8% (36 of 943) of the female veterans in our SARS-CoV-2–positive cohort were found to have G6PD deficiency (Figure 1). G6PD deficiency was documented in 10.8% (418 of 3868) of male veterans in our SARS-COV-2–positive cohort, and most of these veterans were Black (309 of 418 [73.9%]). In comparison, approximately equal numbers of White male veterans (1787) and Black male veterans (1244) were SARS-CoV-2 positive but not G6PD-deficient (Figure 1). Among the male veterans who were G6PD-deficient, only 9% (41 of 454) self-identified as other (not self-identified as Black or White, or self-identified as Asian, Pacific Islander, or American Indian or Alaska Native).

We found a higher prevalence of G6PD deficiency (9.4% [454 of 4811]) (Figure 1) in our SARS-CoV-2–positive cohort than the 2.2% prevalence reported in military statistics (Figure 2). Similarly, 19.9% (309 of 1553) of self-identified Black male veterans in our SARS-CoV-2–positive cohort were G6PD-deficient vs 11.2% of Black men in the military (Figure 2).^39^ Overall, G6PD deficiency was more prevalent in our cohort of veterans who were SARS-CoV-2 positive across all populations assessed, including men and women, than in the general military population (Figure 2).

### G6PD Deficiency and Outcomes From COVID-19 in Black and White Male Veterans

Among younger Black male veterans (aged less than 65 years), the odds of developing severe COVID-19 were greater for veterans with G6PD deficiency than for those without G6PD deficiency (OR 1.47; 95% CI, 1.03-2.09) (Table; eFigure 2 and eTable 2 in Supplement 1). However, the association of G6PD deficiency with severe COVID-19 was not observed in Black male veterans who were older (aged at least 65 years). An approximately 3.6-fold increased likelihood of severe outcomes was, however, observed in the White male veterans with G6PD deficiency aged at least 65 years (n = 37) when compared with White male veterans aged at least 65 years without G6PD deficiency (OR, 3.58; 95% CI, 1.64-7.80) (eFigure 2 in Supplement 1; Table). Although a significant association between G6PD deficiency and severe COVID-19 was not observed in female veterans or in male veterans from other racial ancestry groups, the smaller numbers of veterans in these demographics (eg, female, Asian) preclude firm conclusions regarding these diverse groups of individuals in which G6PD deficiency may still exert a biologic role in COVID-19 severity.

**Table.** Odds of Developing Severe Outcome From COVID-19 Among SARS-CoV-2 Positive US Veterans With G6PD Deficiency^a^

	G6PD deficiency, OR (95% CI)			
	White male veterans	Black male veterans	Other male veterans^b^	All female veterans
Veterans aged <65 y				
No.	31	205	29	33
Crude	1.74 (0.70-4.31)	1.35 (0.97-1.88)	1.29 (0.47-3.53)	1.60 (0.64-3.96)
Adjusted^c^	1.25 (0.47-3.34)	1.47 (1.03-2.09)	0.75 (0.25-2.30)	1.55 (0.61-3.93)
Veterans aged ≥65 y				
No.	37	104	12	3
Crude	3.76 (1.74-8.13)	0.91 (0.60-1.40)	1.60 (0.46-5.61)	0.70 (0.06-8.26)
Adjusted^c^	3.58 (1.64-7.80)	0.95 (0.62-1.47)	1.65 (0.44-6.17)	1.06 (0.08-13.19)

Abbreviations: BMI, body mass index (calculated as weight in kilograms divided by height in meters squared); CKD, chronic kidney disease; G6PD, glucose-6-dehydrogenase; OR, odds ratio.

^a^ Severe outcome defined as (1) in-hospital mortality, (2) hospitalization, (3) intensive care unit admission, or (4) mechanical ventilation.

^b^ Other male veterans included male veterans who did not self-identify as White or Black, or self-identified as Asian, Pacific Islander, or American Indian or Alaska Native.

^c^ Adjusted for age, diabetes, CKD, and BMI. See eFigure 2 in Supplement 1 for graphical representation.

## Discussion

Despite clinical and scientific progress made in the last 3 years with regards to COVID-19 treatments, vaccines and enhanced understanding of its mechanisms of infectivity, the biochemical and physiological reasons behind the disproportionate prevalence of serious complications in minoritized racial and ethnic groups remains unclear. This study is the first to present epidemiologic evidence that putatively link a biologic mechanism of impaired cellular responses in G6PD deficiency to increased COVID-19 severity. This finding is supported by in vitro studies and other studies of SARS/MERS prevalence in patients with G6PD deficiency. Our analysis revealed a strong association between G6PD deficiency and COVID-19 severity, modified by race and age. Although G6PD deficiency was associated with an approximately 1.5-fold increased likelihood of severe outcomes in young Black male veterans, associated increased COVID-19 severity may not be measurable in the older Black male population possibly due to other underlying comorbidities in this population (diabetes and chronic kidney disease, for example) that may already confer a ceiling effect on severity, and that ceiling effect may not be additionally altered (increased) by G6PD deficiency. Contrastingly, in the very small subset of White male veterans with G6PD deficiency, we observed an approximately 3.6-fold increased likelihood of developing severe outcomes from COVID-19 in those aged at least 65 years of age compared with White male veterans aged at least 65 years who were not G6PD-deficient. While differences between Black and White male veterans may be contributed by different G6PD-alleles (eg, G6PD-Mediterranean vs G6PD-A), DOD testing only assesses enzyme activity and does not universally determine genetic variants. Future studies to investigate the potential associations of specific G6PD alleles may be informative.

Although this study did not find significant association in the subpopulations of female veterans and male veterans from other racial backgrounds (eFigure 2 in Supplement 1), our study cohort was not sufficiently powered to evaluate the impact of G6PD deficiency in these groups; thus, additional studies are required to fully assess the possible underlying risk in these populations.

Several potential genetic modifiers related to severity from SARS-CoV-2 infection have been identified, indicating that multiple biochemical and molecular pathways, in addition to G6PD deficiency, are contributing to clinical outcomes.^40,41,42,43^ Genome-wide association studies (GWAS) have identified several genetic risk variants; however, these studies have focused primarily on genetic data from Northern European populations, excluding populations with admixture, an approach that excludes individuals with diverse and/or complex ancestral lineages.^40,42,43,44^ For example, multiple studies have linked variants on chromosome 3p21.31 to worse COVID-19 outcomes; however, the chromosome 3p21.31 allele identified in these studies is most common in European populations and less common in Latino and African American populations.^40,41,42,43,44,45,46^ These and other studies also fail to consider sex in their analyses, limiting the ability to identify specific risks for either sex.^47^ Although these approaches may be informative for larger populations for which genetic data may be more readily available, the exclusion of populations with genetic diversity can contribute to and perpetuate health disparities.

The paucity of biologic and epidemiologic data regarding underserved communities in the United States affected by COVID-19 stem from (with few exceptions) a lack of minoritized racial and ethnic population enrollment in large-scale COVID-19 treatment trials and limited targeted investigations into how genetics and racial ancestry play a role in the inflammatory cascade that is a hallmark of severe infection in COVID-19.^48,49,50,51,52^ Inflammation and oxidative stress are interconnected processes, one being easily induced by the other and both involved in the pathogenesis of COVID-19.^5,7,53^ Glutathione is a crucial antioxidant that mounts a critical defense against oxidative damage from excess reactive oxygen radicals, and is repleted by G6PD. Glutathione augments the innate and the adaptive immunity, conferring protection against bacterial and viral infections.^53^ Separate smaller studies have shown that glutathione was significantly reduced in COVID-19 patients, suggesting that COVID-19 infection either depletes glutathione or that glutathione deficiency or insufficiency may exacerbate outcomes.^53,54^ The results of this present study in combination with other smaller cohort studies and case reports^46,55,56,57,58,59^ underscore the need for targeted investigations into how genetic risk and racial ancestry may contribute to the inflammatory cascade that appears to be a hallmark of severe COVID-19.^49^ These data provide an important initial step in addressing the limited biologic and epidemiologic data regarding underserved communities affected by COVID-19. Avoidance of health care inequities requires careful consideration of the person, their biological sex and gender, ancestry, and community to define underlying risks and to target effective treatment or prevention of disease.

### Strengths and Limitations

This study used a large, national cohort of patients with broad racial and ethnic distribution. Our analysis is among the first and largest with G6PD test records applying a standard retrospective cohort approach to examine the association.

This study also had limitations. Clinical outcomes, and demographic factors, such as race, were self-identified, and comorbidities were obtained through *ICD-10* codes, which may contain misclassification errors. Mortality was limited to in-hospital mortality due to delays in outpatient death reports. Our study was limited to the treatments within VHA health care system, without access to treatments that patients may have received outside the VHA health care. Additional study is warranted by including Medicare and Medicaid information and additional confounding factors, including health care policy messaging, access to care, and usage of nonprescribed and prescribed medications outside of the VHA.

## Conclusions

This cohort study found that younger Black male veterans with G6PD deficiency as a population had an increased likelihood of worse clinical outcomes from SARS-CoV-2 infection. These findings suggest a biologic contribution to these poor outcomes which can be further investigated with targeted efforts in at-risk populations. Appropriate selection of medications and modulation of glutathione levels in patients have the potential to reduce oxidative stress, boost immunity, and reduce the adverse outcomes of COVID-19 infection in the population with G6PD deficiency. This study highlights the need to review and determine possible underlying inherent genetic risks, such as G6PD deficiency, prior to illness or early in treatment course as a strategy to mitigate negative outcomes.

## Notes

Supplement 1. **eTable 1.** Characteristics of SARS-CoV-2-Positive Veterans With or Without G6PD Deficiency **eFigure 1.** COVID+ Veterans With and Without G6PD Deficiency **eFigure 2.** Odds of Developing Severe Outcomes with SARS-CoV-2 Infection in the Presence of G6PD Deficiency **eTable 2.** COVID-19 Outcomes in US Veterans With and Without G6PD Deficiency

Supplement 2.
Data Sharing Statement
