# Supplementary material for: Association of Glucose-6-Phosphate Dehydrogenase Deficiency With Outcomes in US Veterans With COVID-19
Source: JAMA Netw Open. 2023 Mar 29;6(3):e235626. doi: 10.1001/jamanetworkopen.2023.5626 (PMC10061239; doi:10.1001/jamanetworkopen.2023.5626)
Supplement: Supplement 1. — eTable 1. Characteristics of SARS-CoV-2-Positive Veterans With or Without G6PD Deficiency eFigure 1. COVID+ Veterans With and Without G6PD Deficiency eFigure 2. Odds of Developing Severe Outcomes with SARS-CoV-2 Infection in the Presence of G6PD Deficiency eTable 2. COVID-19 Outcomes in US Veterans With and Without G6PD Deficiency [file jamanetwopen-e235626-s001.pdf]

Supplemental Online Content

Elsea SH, Razjouyan J, Lee KM, Lynch JA, Martini S, Pandit LM. Association of glucose-6-phosphate dehydrogenase deficiency with outcomes in US veterans with COVID-19. *JAMA Netw Open.* 2023;6(3):e235626. doi:10.1001/jamanetworkopen.2023.5626

eTable 1. Characteristics of SARS-CoV-2-Positive Veterans With or Without G6PD Deficiency

eFigure 1. COVID+ Veterans With and Without G6PD Deficiency

eFigure 2. Odds of Developing Severe Outcomes with SARS-CoV-2 Infection in the Presence of G6PD Deficiency

eTable 2. COVID-19 Outcomes in US Veterans With and Without G6PD Deficiency

This supplemental material has been provided by the authors to give readers additional information about their work.

© 2023 Elsea SH et al. *JAMA Network Open.*

eTable 1. Characteristics of SARS-CoV-2-Positive Veterans with or without G6PD Deficiency

Study population was limited to patients tested for G6PD deficiency prior to January 1, 2020 and testing positive for SARS-CoV-2 between February 15, 2020 to January 1, 2021. The prevalence of comorbidities commonly associated with risk for severe outcomes due to SARS-CoV-2 infection as of April 15, 2021 are shown. Veterans are grouped by age [<65 y and ≥ 65 y] and self-reported race and sex. The number of veterans (percentage) are shown for each comorbidity, as indicated. Data were extracted and curated from VHA EHR: age (dichotomized into < 65 and ≥ 65 years), BMI (dichotomized into < 30 and ≥ 30 kg/m^2), Deyo-Charlson Comorbidity Index (CCI; dichotomized into < 2 and ≥ 2), racial ancestry, sex, and medical history for the presence of the following: diabetes, chronic kidney disease (CKD), coronary atherosclerosis and other heart disease (CAHD), cardiomyopathy, congestive heart failure (CHF), cardiovascular disease including hypertension (CVD), cancer, chronic obstructive pulmonary disease (COPD), human immunodeficiency virus (HIV), chronic liver disease (CLD), cirrhosis, and alcohol dependency, as indicated by ICD-10 codes. See **Figure 1,** main text.

	US Veterans with Historical Testing for G6PD Deficiency & Positive SARS-Co-V-2 Testing (n = 4811)							
	G6PD Deficiency n = 454				No G6PD Deficiency n = 4,357			
	Men			Women	Men			Women
	White (15.0%)	Black (68.1%)	Other (9.0%)	All (7.9%)	White (41.0%)	Black (28.6%)	Other (9.6%)	All (20.8%)
n	68	309	41	36	1787	1244	419	907
BMI ≥ 30	27(39.7)	136(44.0)	15(36.6)	18(50.0)	724(40.5)	498(40.0)	158(37.7)	331(36.5)
Diabetes	29(42.6)	99(32.0)	13(31.7)	4(11.1)	376(21.0)	495(39.8)	61(14.6)	81(8.9)
CKD	20(29.4)	71(23.0)	10(24.4)	1(2.8)	214(12.0)	267(21.5)	31(7.4)	31(3.4)
CAHD	24(35.3)	47(15.2)	8(19.5)	2(5.6)	274(15.3)	227(18.2)	32(7.6)	29(3.2)
Cardiomyopathy	7(10.3)	15(4.9)	4(9.8)	0	56(3.1)	76(6.1)	8(1.9)	3(0.3)
CHF	10(14.7)	37(12.0)	4(9.8)	0	133(7.4)	142(11.4)	16(3.8)	11(1.2)
CVD	34(50.0)	105(34.0)	12(29.3)	5(13.9)	441(24.7)	451(36.3)	58(13.8)	83(9.2)
Cancer	24(35.3)	78(25.2)	6(14.6)	6(16.7)	298(16.7)	290(23.3)	33(7.9)	82(9.0)
COPD	12(17.6)	39(12.6)	4(9.8)	3(8.3)	242(13.5)	249(20.0)	27(6.4)	27(3.0)
HIV	12(17.6)	46(14.9)	5(12.2)	2(5.6)	138(7.7)	271(21.8)	29(6.9)	18(2.0)
CLD	12(17.6)	27(8.7)	6(14.6)	4(11.1)	137(7.7)	108(8.7)	20(4.8)	23(2.5)
Cirrhosis	5(7.4)	11(3.6)	2(4.9)	0	48(2.7)	52(4.2)	9(2.1)	1(0.1)
Alcohol Dep.	6(8.8)	39(12.6)	6(14.6)	3(8.3)	127(7.1)	172(13.8)	23(5.5)	41(4.5)
CCI ≥ 2	47(69.1)	163(52.8)	18(43.9)	8(22.2)	635(35.5)	749(60.2)	91(21.7)	123(13.6)
Severe Outcome[a]	34(50.0)	126(40.8)	12(29.3)	7(19.4)	375(21.0)	473(38.0)	78(18.6)	122(13.5)

© 2023 Elsea SH et al. *JAMA Network Open.*

	G6PD Deficiency				No G6PD Deficiency			
**Age < 65 years (n = 3,583)**								
	Men			Women	Men			Women
	White	Black	Other	All	White	Black	Other	All
n	31	205	29	33	1288	779	359	859
BMI ≥ 30	15(48.4)	101(49.3)	10(34.5)	18(54.5)	520(40.4)	359(46.1)	142(39.6)	312(36.3)
Diabetes	7(22.6)	51(24.9)	8(27.6)	3(9.1)	127(9.9)	235(30.2)	29(8.1)	55(6.4)
CKD	5(16.1)	30(14.6)	3(10.3)	1(3.0)	52(4.0)	104(13.4)	13(3.6)	18(2.1)
CAHD	3(9.7)	18(8.8)	2(6.9)	2(6.1)	55(4.3)	79(10.1)	9(2.5)	18(2.1)
Cardiomyopathy	0	7(3.4)	1(3.4)	0	14(1.1)	31(4.0)	4(1.1)	3(0.3)
CHF	0	14(6.8)	0	0	24(1.9)	44(5.6)	3(0.8)	4(0.5)
CVD	5(16.1)	50(24.4)	3(10.3)	5(15.2)	122(9.5)	182(23.4)	23(6.4)	61(7.1)
Cancer	4(12.9)	25(12.2)	2(6.9)	6(18.2)	87(6.8)	96(12.3)	14(3.9)	67(7.8)
COPD	2(6.5)	14(6.8)	0	3(9.1)	58(4.5)	86(11.0)	7(1.9)	15(1.7)
HIV	11(35.5)	32(15.6)	3(10.3)	2(6.1)	110(8.5)	191(24.5)	25(7.0)	18(2.1)
CLD	5(16.1)	17(8.3)	3(10.3)	4(12.1)	76(5.9)	61(7.8)	10(2.8)	19(2.2)
Cirrhosis	2(6.5)	7(3.4)	1(3.4)	0	13(1.0)	23(3.0)	2(0.6)	1(0.1)
Alcohol Dep.	2(6.5)	26(12.7)	3(10.3)	3(9.1)	83(6.4)	108(13.9)	21(5.8)	41(4.8)
CCI ≥ 2	13(41.9)	81(39.5)	8(27.6)	8(24.2)	230(17.9)	365(46.9)	44(12.3)	92(10.7)
Severe Outcome[a]	6(19.4)	67(32.7)	5(17.2)	6(18.2)	155(12.0)	206(26.4)	50(13.9)	102(11.9)
**Age ≥ 65 years (n = 1228)**								
	G6PD Deficiency				No G6PD Deficiency			
	Men			Women	Men			Women
	White	Black	Other	All	White	Black	Other	All
n	37	104	12	3	499	465	60	48
BMI ≥ 30	12(32.4)	35(33.7)	5(41.7)	0	204(40.9)	139(29.9)	16(26.7)	19(39.6)
Diabetes	22(59.5)	48(46.2)	5(41.7)	1(33.3)	249(49.9)	260(55.9)	32(53.3)	26(54.2)
CKD	15(40.5)	41(39.4)	7(58.3)	0	162(32.5)	163(35.1)	18(30.0)	13(27.1)
CAHD	21(56.8)	29(27.9)	6(50.0)	0	219(43.9)	148(31.8)	23(38.3)	11(22.9)
Cardiomyopathy	7(18.9)	8(7.7)	3(25.0)	0	42(8.4)	45(9.7)	4(6.7)	0
CHF	10(27.0)	23(22.1)	4(33.3)	0	109(21.8)	98(21.1)	13(21.7)	7(14.6)
CVD	29(78.4)	55(52.9)	9(75.0)	0	319(63.9)	269(57.8)	35(58.3)	22(45.8)
Cancer	20(54.1)	53(51.0)	4(33.3)	0	211(42.3)	194(41.7)	19(31.7)	15(31.3)
COPD	10(27.0)	25(24.0)	4(33.3)	0	184(36.9)	163(35.1)	20(33.3)	12(25.0)
HIV	1(2.7)	14(13.5)	2(16.7)	0	28(5.6)	80(17.2)	4(6.7)	0
CLD	7(18.9)	10(9.6)	3(25.0)	0	61(12.2)	47(10.1)	10(16.7)	4(8.3)
Cirrhosis	3(8.1)	4(3.8)	1(8.3)	0	35(7.0)	29(6.2)	7(11.7)	0
Alcohol Dep.	4(10.8)	13(12.5)	3(25.0)	0	44(8.8)	64(13.8)	2(3.3)	0
CCI ≥ 2	29(78.3)	82(78.8)	10(83.3)	0	405(81.2)	384(82.6)	47(78.3)	31(64.6)
Severe Outcome[a]	28(75.7)	59(56.7)	7(58.3)	1(33.3)	220(44.1)	268(57.6)	28(46.7)	20(41.7)

[a]Severe outcome defined as any of the following: (i) in-hospital mortality, (ii) hospitalization, (iii) intensive care unit admission or (iv) mechanical ventilation. Abbreviations: Other = males not self-identified as White nor Black, or self-identified as Asian, Pacific Islander or Native American; BMI = body mass index; CAHD = coronary artery heart disease; CKD = chronic kidney disease; CLD = chronic liver disease; COPD = chronic obstructive pulmonary disease; Dep=dependence; CHF = congestive heart failure; CVD = cardiovascular disease; HIV = human immunodeficiency virus; CCI = Charlson Comorbidity Index

© 2023 Elsea SH et al. *JAMA Network Open.*

eFigure 1. COVID+ Veterans with and without G6PD Deficiency. Heatmap illustrates the prevalence of comorbidities commonly associated with risk for severe outcomes due to SARS-CoV-2 infection (n = 4811). Percentages for each comorbidity present in SARS-CoV-2 positive veterans with or without G6PD deficiency are shown and represented in color by the heatmap scale shown on the right. Data represent the entire cohort (n=4811) stratified by G6PD deficiency status, race, and sex. Cohort is not stratified by age in this representation.

COVID+ Veterans[a]

	G6PD Deficient				Not G6PD Deficient			
	WM	BM	OM	F	WM	BM	OM	F
% Total Population	1.4	6.4	0.9	0.8	37.1	25.9	8.7	18.9
% G6PD Sub-Cohort	15.0	68.1	9.0	7.9	41.0	28.6	9.6	20.8
BMI ≥ 30	39.7	44.0	36.6	50.0	40.5	40.4	37.7	36.5
Diabetes	42.6	32.0	31.7	11.1	21.0	39.8	14.6	8.9
CKD	29.4	23.0	24.4	2.8	12.0	21.5	7.4	3.4
CAHD	35.3	15.2	19.5	5.6	15.3	18.2	7.6	3.2
Cardiomyopathy	10.3	4.9	9.8	0.0	3.1	6.1	1.9	0.3
CHF	14.7	12.0	9.8	0.0	7.4	11.4	3.8	1.2
CVD	50.0	34.0	29.3	13.9	24.7	36.3	13.8	9.2
Cancer	35.3	25.2	14.6	16.7	16.7	23.3	7.9	9.0
COPD	17.6	12.6	9.8	8.3	13.5	20.0	6.4	3.0
HIV	17.6	14.9	12.2	5.6	7.7	21.8	6.9	2.0
CLD	17.6	8.7	14.6	11.1	7.7	8.7	4.8	2.5
Cirrhosis	7.4	3.6	4.9	0.0	2.7	4.2	2.1	0.1
Alcohol Dep.	8.8	12.6	14.6	8.3	7.1	13.8	5.5	4.5
CCI ≥ 2	69.1	52.8	43.9	22.2	35.5	60.2	21.7	13.6
Severe outcome[b]	50.0	40.8	29.3	19.4	21.4	38.0	18.6	13.5

% scale: 0, 20, 40, 60, 80, 100

[a]Study population, n=4811; [b]Severe outcomes include (i) in-hospital mortality, (ii) hospitalization, (iii) intensive care unit admission or (iv) mechanical ventilation; WM= White male; BM = Black male; OM = Other male not self-identified as White nor Black, or self-identified as Asian, Pacific Islander or Native American; F = All females; BMI = body mass index; CAHD = coronary artery heart disease; CKD = chronic kidney disease; CLD = chronic liver disease; COPD = chronic obstructive pulmonary disease; Dep=dependence; CHF = congestive heart failure; CVD = cardiovascular disease; HIV = human immunodeficiency virus; CCI = Charlson Comorbidity Index

© 2023 Elsea SH et al. *JAMA Network Open.*

eFigure 2. Odds of Developing Severe Outcomes with SARS-CoV-2 Infection in the Presence of G6PD Deficiency. Adjusted odds ratios (OR) for severe disease in the presence of G6PD deficiency, stratified by self-reported race and sex, and adjusted for age, diabetes, CKD, and BMI are shown. Black men <65 years of age (black filled squares) and White men ≥65 years of age (black filled circles) have increased odds of severe outcomes, demonstrating 1.47X for young Black men and 3.56X for older White men, respectively. Significance is indicated by filled symbols. Open symbols are not significant. G6PD = glucose-6-phoshate dehydrogenase; Black males <65 years, n= 984; Black males >65 y, n=569; White males <65 y, n=1319; White males >65 y, n=536; Other males <65y, n= 388; Other males >65 y, n=72; All females <65 y, n= 892; All females >65 y, n= 51. Other = Males not self-identified as White or Black, or self-identified as Asian, Pacific Islander, or Native American.

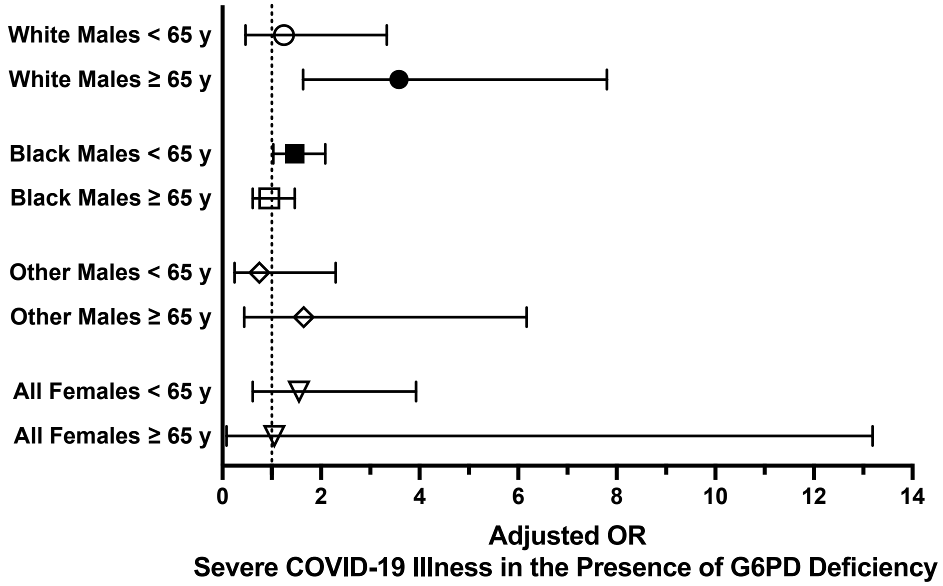

© 2023 Elsea SH et al. *JAMA Network Open.*

eTable 2. COVID-19 Outcomes in US Veterans with and without G6PD Deficiency

	G6PD Deficiency				No G6PD Deficiency			
	Men (n = 418)			Women (n=36)	Men (n=3450)			Women (n=907)
**Outcomes**	**White**	**Black**	**Other**	**All**	**White**	**Black**	**Other**	**All**
**Hosp total (%)**	**34(50.0)**	**126(40.8)**	**12(29.3)**	**7(19.4)**	**375(21.0)**	**473(38.0)**	**78(18.6)**	**122(13.5)**
- ICU	18(26.5)	60(19.4)	3(7.3)	2(5.6)	185(10.4)	224(18.0)	48(11.5)	68(7.5)
- Vent	9(13.2)	23(7.4)	2(4.9)	1(2.8)	66(3.7)	98(7.9)	12(2.9)	11(1.2)
- Death	8(11.8)	14(4.5)	3(7.3)	1(2.8)	43(2.4)	81(6.5)	5(1.2)	5(0.6)
**age < 65 years**								
**Hosp total (%)**	**6(19.4)**	**67(32.7)**	**5(17.2)**	**6(18.2)**	**155(12.0)**	**205(26.3)**	**50(13.9)**	**102(11.9)**
- ICU	2(6.5)	35(17.1)	0(0.0)	2(6.1)	84(6.5)	91(11.7)	31(8.6)	58(6.8)
- Vent	1(3.2)	12(5.9)	0(0.0)	1(3.0)	20(1.6)	37(4.7)	4(1.1)	7(0.8)
- Death	0(0.0)	4(2.0)	0(0.0)	1(3.0)	3(0.2)	16(2.1)	0(0.0)	1(0.1)
**age ≥ 65 years**								
**Hosp total (%)**	**28(75.7)**	**59(56.7)**	**7(58.3)**	**1(33.3)**	**220(44.1)**	**268(57.6)**	**28(46.7)**	**20(41.7)**
- ICU	16(43.2)	25(24.0)	3(25.0)	0(0.0)	101(20.2)	133(28.6)	17(28.3)	10(20.8)
- Vent	8(21.6)	11(10.6)	2(16.7)	0(0.0)	46(9.2)	61(13.1)	8(13.3)	4(8.3)
- Death	8(21.6)	10(9.6)	3(25.0)	0(0.0)	40(8.0)	65(14.0)	5(8.3)	4(8.3)

Abbreviations: Hosp = hospital; ICU – intensive care unit; Vent – ventilator; Other = Males not self-identified as White nor Black, or self-identified as Asian, Pacific Islander or Native American

© 2023 Elsea SH et al. *JAMA Network Open.*
