# Supplementary material for: Association of Glucose-6-Phosphate Dehydrogenase Deficiency With Outcomes in US Veterans With COVID-19
Source: JAMA Netw Open. 2023 Mar 29;6(3):e235626. doi: 10.1001/jamanetworkopen.2023.5626 (PMC10061239; doi:10.1001/jamanetworkopen.2023.5626)
Supplement: Supplement 2. — Data Sharing Statement [file jamanetwopen-e235626-s002.pdf]

Data Sharing Statement

Elsea. Association of Glucose-6-Phosphate Dehydrogenase Deficiency With Outcomes in US Veterans With COVID-19. *JAMA Netw Open*. Published March 29, 2023. doi:10.1001/jamanetworkopen.2023.5626

Data
Data available: No

Additional Information
Explanation for why data not available: Individual patient data from veterans are not available due department of veteran affairs privacy, but raw data from the entire dataset is available in the supplement at at request.
